# Does Vertebroplasty Affect Radiation Dose Distribution?: Comparison of Spatial Dose Distributions in a Cement-Injected Vertebra as Calculated by Treatment Planning System and Actual Spatial Dose Distribution

**DOI:** 10.1155/2012/571571

**Published:** 2012-04-10

**Authors:** Atsushi Komemushi, Noboru Tanigawa, Shuji Kariya, Rie Yagi, Miyuki Nakatani, Satoshi Suzuki, Akira Sano, Koshi Ikeda, Keita Utsunomiya, Yoko Harima, Satoshi Sawada

**Affiliations:** Department of Radiology, Kansai Medical University, 10-15 Fumizono-cho, 570-8507 Moriguchi, Osaka, Japan

## Abstract

*Purpose*. To assess differences in dose distribution of a vertebral body injected with bone cement as calculated by radiation treatment planning system (RTPS) and actual dose distribution. *Methods*. We prepared two water-equivalent phantoms with cement, and the other two phantoms without cement. The bulk density of the bone cement was imported into RTPS to reduce error from high CT values. A dose distribution map for the phantoms with and without cement was calculated using RTPS with clinical setting and with the bulk density importing. Actual dose distribution was measured by the film density. Dose distribution as calculated by RTPS was compared to the dose distribution measured by the film dosimetry. *Results*. For the phantom with cement, dose distribution was distorted for the areas corresponding to inside the cement and on the ventral side of the cement. However, dose distribution based on film dosimetry was undistorted behind the cement and dose increases were seen inside cement and around the cement. With the equivalent phantom with bone cement, differences were seen between dose distribution calculated by RTPS and that measured by the film dosimetry. *Conclusion*. The dose distribution of an area containing bone cement calculated using RTPS differs from actual dose distribution.

## 1. Introduction

 Pain due to bone metastasis is one of the prevalent complications of cancer [1–5]. Though the value of vertebroplasty for osteoporotic fracture is discussed [6, 7], even now Percutaneous vertebroplasty has been considered one of good therapeutic options for alleviating pain caused by malignant vertebral tumors, such as multiple myeloma and metastatic vertebral tumor [8–15]. Radiotherapy is gold standard treatment for pain associated with metastatic bone tumor. The National Comprehensive Cancer Network guidelines [16] and Ontario guidelines [17] recommend radiotherapy for alleviating the pain associated with bone metastasis.

 In the treatment of metastatic spinal bone tumor, combination therapy consisting of vertebroplasty and radiotherapy has been performed [18, 19]. In future, radiotherapy combined with percutaneous vertebroplasty is expected to be performed more frequently.

Bone cement used in percutaneous vertebroplasty contains about 30% barium, a radio-opaque agent [10–13]. The X-ray absorption value of cement containing about 30% barium is very high (computed tomography (CT) value: 1600–3000 HU). When planning three-dimensional radiotherapy using CT images following percutaneous vertebroplasty, bone cement containing barium in vertebral bodies may affect the dose distribution of radiotherapy, but no basic data are available regarding the effects of bone cement on dose distribution. The objective of the present study was to clarify the effects of bone cement containing barium in vertebral bodies on dose distribution and the differences in dose distribution calculated using a radiation treatment planning system (RTPS) and actual dose distribution.

## 2. Materials and Methods

### 2.1. Phantom Preparation

 Four water-equivalent phantoms (Toughwater Phantom, 1-cm thickness, 1.018 g/cm^3^, Kyoto Kagaku CO., Kyoto, Japan) were prepared by tracing the shape of the anthropomorphic phantom (RANDO phantom, Kyoto Kagaku CO., Kyoto, Japan) at the second lumbar vertebra (between number 23 and 24) according to ICRU Report number 44.

**Figure 1:**
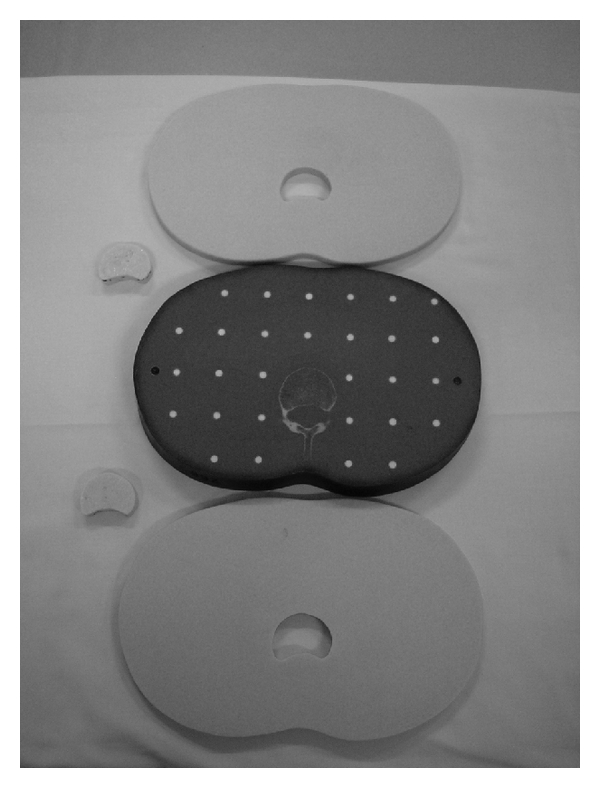
The middle phantom is a number 23 RANDO phantom. The upper and lower phantoms are water-equivalent phantoms modeled after the anthropomorphic phantom, and a test hole corresponding to the second lumbar vertebral body has been hollowed out. Testing sample such as PMMA and Toughwater Phantom is set in the hole.

 With two water-equivalent phantoms, the area corresponding to the second lumbar vertebral body was scooped out as shown in Figure 1. Twenty grams of methylmethacrylate powder (Osteobond copolymer bone cement; Zimmer, Warsaw, IN, USA) was mixed with 5 grams of barium sulfate powder (Horii Pharmaceutical, Osaka, Japan). Ten milliliters of liquid methylmethacrylate monomer was added to the resulting powder, and the mixture was blended to a toothpaste-like consistency to produce polymethylmethacrylate (PMMA). The resulting polymethylmethacrylate was then poured into the hollowed-out area of the water-equivalent phantoms and then cured to prepare water-equivalent phantoms with bone cement. With the remaining two water-equivalent phantoms, bone cement was not poured (control).

### 2.2. Calculation of Dose Distribution

 The water-equivalent phantom modeled after the second lumbar vertebra was subjected to CT (Asteion 4; Toshiba Medical Systems, Tokyo, Japan) (imaging condition: 120 kV; 250 mA; 5 mm slice thickness). CT image data was transferred to a radiation treatment planning system (Eclipse Treatment Planning System, version 8.1; Varian Medical Systems, CA, USA) to plan radiotherapy. An isocenter for radiotherapy was set inside the spinal cord on a target slice, and a 10 cm × 10 cm irradiation field was established around the isocenter for posterior single field irradiation. The reference point for radiotherapy was set at the isocenter. Using 4-MV X-ray, 1 Gy of radiation was irradiated. Using the treatment planning system with clinical setting, a dose distribution map was prepared for the water-equivalent phantoms with and without bone cement.

 Additionally, we imported the bulk density (1.303 g/cm^3^) of the bone cement into the treatment planning system to reduce error from high CT values, and a dose distribution map was again prepared for the water-equivalent phantoms with and without bone cement.

### 2.3. Measurement of Dose Distribution by the Film Dosimetry

**Figure 2:**
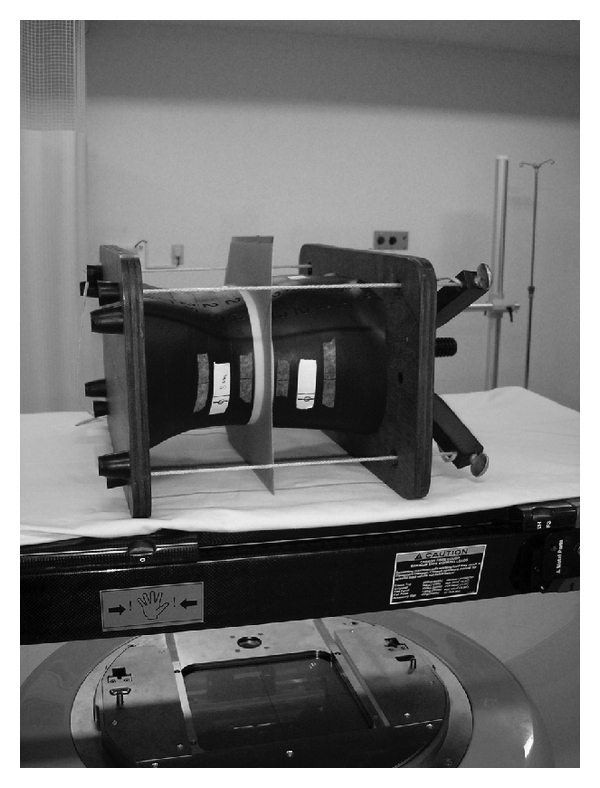
A film (EDR2) is located between two water-equivalent phantoms. They were firmly set in the anthropomorphic phantoms, and radiation was irradiated.

 A film (EDR2; Eastman Kodak Co., Rochester, NY, USA) was placed between two water-equivalent phantoms (Figure 2). Under the same conditions for measuring dose distribution using RTPS, linear accelerator (clinac21EX; Varian Medical Systems, CA, USA) was used to irradiate 1 Gy of 4-MV X-ray. As a reference point, 100% dose was irradiated. Films were developed and read using a universal flat-bed scanner (ES-10000G; Epson, Nagano, Japan), and a dose distribution analysis system (DD-System, DD-Analysis version 8.0, DD-IMRT version 8.0 and DD-Scan version 3.0; R-Tech, Tokyo, Japan) was used to measure dose distribution based on the film density [20]. Dose distribution was assessed by defining the isocenter on each film as the origin (coordinate: 0), left-right direction as the *X*-axis and anteroposterior direction as the *Y*-axis.

 Effects of bone cement on irradiation as assessed by the film dosimetry were investigated by comparing dose distribution between water-equivalent phantoms with and without bone cement. Dose distribution as calculated by RTPS was compared to the dose distribution measured by the film dosimetry.

## 3. Results

### 3.1. Dose Distribution Calculated by RTPS

**Figure 3:**
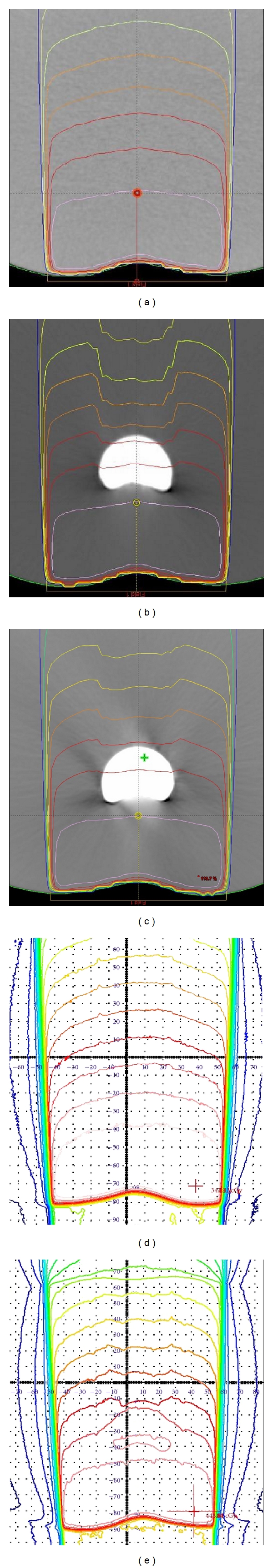
The calculated dose distribution maps using RTPS and the dose distribution maps measured by film dosimetry. (a) with the phantom without bone cement, the calculated dose distribution was undistorted. (b) with the phantom with bone cement, using RTPS with clinical setting, a depression in the isodose curve was seen for areas corresponding to inside the cement and the ventral side of the cement. (c) with the phantom with bone cement, using RTPS with importing the bulk density of the bone cement, dose inside the cement was the same as in the surrounding area. A depression in the isodose curve was seen in the ventral side of the cement however, the distortion with importing the bulk density of the bone cement (c) was less than with clinical setting (b). (d) with the phantom without bone cement, no distortion and no difference in the isodose curve were seen between calculated dose distribution (a) and measured dose distribution (d). (e) with the phantom with bone cement, a dose distribution map drawn by the film-based dose distribution analysis system. Dose increases were seen within and around bone cement. No significant dose decrease was seen behind bone cement.

 For the water-equivalent phantom without bone cement, dose distribution calculated using RTPS both with clinical setting and with importing the bulk density of the bone cement was even and undistorted (Figure 3(a)).

 For the water-equivalent phantom with bone cement, dose distribution calculated with clinical setting was distorted for the areas corresponding to inside the cement and on the ventral side of the cement. In other words, dose inside the cement was lower than in the surrounding area. Dose for the ventral side (the side through which radiation had passed through the cement) was lower than that for the dorsal side (the side through which radiation had yet to pass through the cement). A dose distribution map showed that dose was lower for the area after the cement (Figure 3(b)).

 For the water-equivalent phantom with bone cement, dose distribution calculated with importing the bulk density of the bone cement was not distorted for the areas corresponding to inside the cement but distorted on the ventral side of the cement. Dose inside the cement was the same as in the surrounding area. Dose for the ventral side was lower than that for the dorsal side; however, the distortion with importing the bulk density of the bone cement was less than with clinical setting (Figure 3(c)).

### 3.2. Analysis by the Film-Based Dose Distribution Analysis System

 For the equivalent phantom without bone cement, dose distribution was undistorted (Figure 3(d)). For the equivalent phantom with bone cement, dose increases were seen inside cement and around the cement (Figure 3(e)).

In the *Y*-axis direction, between the equivalent phantom with bone cement and the equivalent phantom without bone cement, differences existed inside and outside the cement. Dose increase inside bone cement was 8.94 cGy ± 2.78 (maximum, 15.64 cGy). Outside bone cement, dose increase was found only before bone cement, but no dose increase was seen after cement. Area with dose increase outside bone cement was 2.33 mm ± 1.03 (maximum, 3.0 mm). Dose increase outside bone cement was 1.35 cGy ± 0.68 (maximum, 2.68 cGy).

Dose distribution was analyzed in the *X*-axis direction at the maximum width of bone cement. Dose increases were seen inside and outside bone cement. Dose increase inside bone cement was 12.19 cGy ± 2.27 (maximum, 16.73 cGy). Area with dose increase outside bone cement was 11.50 mm ± 2.66 (maximum, 15.0 mm). Dose increase outside bone cement was 3.65 cGy ± 1.54 (maximum, 8.35 cGy).

### 3.3. Comparison of Dose Distribution as Calculated by Treatment Planning and Film-Based Dose Distribution Analysis

 With the equivalent phantom without bone cement, no difference and no distortion were seen between dose distribution calculated by RTPS and that measured using the film-based dose distribution analysis system.

 With the equivalent phantom with bone cement, differences were seen between dose distribution calculated by RTPS and that measured by the film-based dose distribution analysis system. Dose at the bone cement as calculated by RTPS with clinical setting was lower than that in the surrounding area, and with importing the bulk density of the bone cement was similar to that in the surrounding area, but dose at the bone cement as calculated by the film dosimetry was higher than that in the surrounding area. Dose for the area after bone cement was lower than that for adjacent area according to RTPS with clinical setting, but the dose distribution calculated by RTPS with importing the bulk density of the bone cement and the dose distribution measured using the dose distribution analysis system did not confirm any dose reduction after bone cement.

## 4. Discussion

 Three-dimensional radiotherapy planning is based on CT, but when performing radiotherapy following percutaneous vertebroplasty, bone cement containing barium in vertebral bodies can affect the dose distribution of radiotherapy. The present basic study using water-equivalent phantoms was performed to clarify the effects of bone cement containing barium in vertebral bodies by percutaneous vertebroplasty on dose distribution during radiotherapy and to ascertain differences between dose distribution calculated by RTPS and actual dose distribution.

 The RTPS cannot handle the conversion from these high CT values to electron densities, and the barium could have generated CT artifacts making the contouring of the area with bone cement less accurate. Therefore, the authors imported the bulk density of the bone cement into the RTPS. By assuming that the bulk density of the cement is also the electron density relative to water, probably a much more realistic dose distribution will be obtained. This is in fact the procedure recommended for other situations where high-*Z* materials, such as hip prostheses, cause CT artefacts, as outlined in Report of the AAPM Radiation Therapy Committee Task Group 63 [21].

### 4.1. Dose Increase Inside and Around Bone Cement

 In our study, dose increases were seen within and before and to the lateral sides of bone cement. Dose increase could have been caused by scattering of radiation by the cement. However, no significant dose increase was confirmed after cement. In particular, dose increase before cement could have been caused by backscattering of irradiation by the cement. The distance from vertebral bodies to spinal cord is about 2 mm. In the present study, the area with dose increase outside bone cement caused by back scattering of irradiation was 2.33 mm, and the degree of dose increase outside bone cement was 1.35 cGy. Therefore, spinal cord dose can appear to exceed planned dose.

### 4.2. Dose Distribution

 In treatment planning using RTPS, the dose decrease was calculated after passing through bone cement. However, no significant dose decrease was seen by the film dosimetry.

 In radiotherapy for metastatic bone tumors in the thoracic and lumbar vertebrae, posterior single-field irradiation is generally performed. When performing posterior single field irradiation for thoracolumbar vertebrae, caution must be exercised on spinal cord dose. As reported by Emami et al. [22], the dose resulting in a 5% probability of this devastating complication such as radiation myelitis within 5 years of treatment (TD 5/5) has been estimated at 50 Gy, and 45 Gy has commonly been used as a maximum dose in clinical practice. The cauda equina, consisting of spinal nerve roots, has been found to have a slightly higher tolerance, with an estimated TD 50/5 of 60 Gy [22]. In the present study, there was back scattering, but spinal cord dose can not appear to extremely exceed planned dose. Hence, even with vertebral bodies with bone cement placed by percutaneous vertebroplasty, when performing radiotherapy by posterior single field irradiation, spinal cord dose is a little affected.

 In recent years, new radiotherapeutic techniques such as intensity-modulated radiation therapy (IMRT) and body stereotactic radiotherapy have been performed [23–26]. When performing radiotherapy for paravertebral tumors around vertebral bodies (e.g., lung cancer, esophageal cancer, and pancreatic cancer) following bone cement injection, if bone cement exists within an irradiation field, then RTPS will calculate radiation dose after the bone cement as low, but in reality, since bone cement does not affect radiation dose, a higher dose of radiation may be delivered over a broader area than expected.

 The difference between the dose distribution calculated using RTPS and the dose distribution measured by the film dosimetry could be explained as follows. First, bone cement has as high as 30% barium concentration. The RTPS did not have data for the effects of an artificial material (bone cement containing barium) on dose distribution. Secondly, bone cement containing barium could have generated CT artifacts. Hence, if bone cement exists within an irradiation field during treatment planning, expected dose distributions may differ from actual dose distribution. Importing the bulk density of the bone cement into RTPS could decrease the difference between the dose distribution calculated using RTPS and the dose distribution measured by the film dosimetry.

 In conclusion, the dose distribution of an area containing bone cement calculated using RTPS differs from actual dose distribution.
